# National profile of foot orthotic provision in the United Kingdom, part 2: podiatrist, orthotist and physiotherapy practices

**DOI:** 10.1186/s13047-018-0250-9

**Published:** 2018-03-20

**Authors:** C. J. Nester, A. Graham, A. Martinez-Santos, A. E. Williams, J. McAdam, V. Newton, D. Sweeney, D. Walker

**Affiliations:** School of Health Sciences, Brian Blatchford Building, University of Salford, Salford, UK

## Abstract

**Background:**

A national survey recently provided the first description of foot orthotic provision in the United Kingdom. This article aims to profile and compare the foot orthoses practice of podiatrists, orthotists and physiotherapists within the current provision.

**Method:**

Quantitative data were collected from podiatrists, orthotists and physiotherapists via an online questionnaire. The topics, questions and answers were developed through a series of pilot phases. The professions were targeted through electronic and printed materials advertising the survey. Data were captured over a 10 month period in 2016. Differences between professions were investigated using Chi squared and Fischer’s exact tests, and regression analysis was used to predict the likelihood of each aspect of practice in each of the three professions.

**Results:**

Responses from 357 podiatrists, 93 orthotists and 49 physiotherapists were included in the analysis. The results reveal statistically significant differences in employment and clinical arrangements, the clinical populations treated, and the nature and volume of foot orthoses caseload.

**Conclusion:**

Podiatrists, orthotists and physiotherapists provide foot orthoses to important clinical populations in both a prevention and treatment capacity. Their working context, scope of practice and mix of clinical caseload differs significantly, although there are areas of overlap. Addressing variations in practice could align this collective workforce to national allied health policy.

**Electronic supplementary material:**

The online version of this article (10.1186/s13047-018-0250-9) contains supplementary material, which is available to authorized users.

## Background

Provision of foot orthoses falls within the scope of practice of a range of health care professionals and a national survey of the foot orthotic practice of podiatrists, orthotists and physiotherapists was recently reported for the first time [1]. That a range of professions are involved is positive since it encourages delivery of suitable care at various points within a health care system, which might better suit patients. It also allows the historical roles of different professions, and nuances of differences in education, training and practice philosophies to influence the wider orthotic practice sector. This too is positive since it facilitates other areas of knowledge and experience influencing orthotic practice, and this should be an environment within which innovation and development of practice can flourish.

Our previous article that reported on a survey of foot orthoses practice at a national level [1] also identified differences in almost all aspects of practice between podiatrists, orthotists and physiotherapists. This may relate to prior training, scope or nature of practice and work place, or the different contractual arrangements for the professions. It may also relate to the degree of scaling of specialism towards the foot (podiatrists) and orthoses (orthotists), versus the whole body (physiotherapists and orthotists). This resonates with recent policy priorities to support integration of areas of practice in ways that are patient centred rather than beholden to historical service or professional boundaries [2].

The aim of this article is to profile and compare the foot orthotic practice of podiatrists, orthotists and physiotherapists in the United Kingdom. It is important to understand variation in practice to inform education and training, to consider needs for standardisation of practice (e.g. related to quality assurance), and in managing future workplace planning (e.g. the need for greater orthotic skills in health care workforce) [3]. With this data, individual practitioners would be able to better reflect on their own practice, and contextualise it within their own profession and allied professions. Managers would be able to contextualise the practice of their staff and the different service models that relate to the three professions. Researchers would be able to understand the extent to which their research applies to the practice of the three professions. Industry would be able to understand differences between potential client bases. Finally, national policy makers will be able to gauge, for the first time, the different practices of the professions concerned with foot orthotic use nationally and look to understand these in the context of national policy related to allied health professionals [2] and health priorities.

## Methods

A questionnaire suitable for online completion was used to capture the foot orthotic practice of podiatrists, orthotists and physiotherapists (same data set as [1]). The survey was approved by the institutional ethics committee (HSCR14/125) and online data capture provided a means of securing informed consent.

### Survey development and piloting

Four academic staff with professional backgrounds in orthotics and podiatry practice and services, and foot health research, worked with seven clinical podiatry and orthotist practitioners to identify topics for the survey, sample questions and responses, and identify appropriate terminology. This produced a draft survey fit for pilot testing with a wider audience.

The draft survey questionnaire was implemented within the Bristol Online Survey platform (https://www.onlinesurveys.ac.uk/) and piloted through two iterations with orthotists, podiatrists, and physiotherapists. Questions were then rationalised down from 90 to 60 based on feedback. The final survey consisted of 5 main sections with 60 questions in total (appendix A).

### Sampling

Invitations to participate were distributed between January and June 2016. All correspondence was standardised in terms of language and provided a hyperlink to the online survey. Press releases were provided for publications of the Society of Chiropodists and Podiatrists, Chartered Society of Physiotherapists, and the British Association of Prosthetists and Orthotists. Specialist interest groups were also targeted and 20 commercial suppliers of orthoses related materials and products (to podiatrists, orthotists, and physiotherapists) were contacted. They sent out electronic or printed notifications to customers. These were also distributed at professional body conferences.

### Data collection

The questionnaire comprised a mixture of open-ended, closed-ended dichotomous, contingency, nominal and ordinal polytomous questions were used to reduce the risk of missing data [4, 5]. It was anonymous, self-administered and conducted on the Bristol Online Survey website. The survey was open from January 1st to October 31st 2016.

### Data analysis

All data were statistically analysed with SPSS version 21.0 (IBM Corp, Somers, NY, USA). The percentage of podiatrists, orthotists and physiotherapists who responded to each question was calculated to describe responses for each profession. For responses to fixed choice questions, a Chi squared test was performed (significance level of 95%) to identify significant differences between professions. However, a Fisher’s exact test was used in preference to Chi squared if, based on crosstab of the data, the number of responses was less than 5 in more than 20% of the data [6]. Multiple logistic regression tests (deriving the Odds Ratio Exp(B), zero-infinity) were then completed on each significant response to indicate the scale and direction of the differences between pairs of professions. This provided an estimate of the likelihood of a specific response occurring in each profession versus each of the other two professions. A ratio of one means there is equivalent likelihood of the response to the question in the two professions concerned, more than one means greater likelihood of the response in one profession compared to the other, and vice versa for the other profession if the ratio is less than one.

## Results

A total of 512 responses were received and 13 were removed (1 patient, 1 student, 1 occupational therapist and 10 from outside the United Kingdom). The data (499 responses) comprised responses from 357 podiatrists, 93 orthotists and 49 physiotherapists.

### Work place and training

The main work place for foot orthoses practice was the National Health Service (55.2% podiatrists, 51.0% physiotherapists and 45.2% for orthotists) (Table 1, also see Additional file 1: Table S1). Outside of the National Health Service, 28.9% of podiatrists were self-employed and these responders were 6.8 times more likely to be self-employed than orthotists, and 2.3 times more likely than physiotherapists. Orthotists were the profession most likely to be working in a private company providing National Health Service services (36.6% of orthotist responders, versus < 2% for each of podiatrists and physiotherapists).

**Table 1:** Practice context (percentage of responders in each profession and results of logistic regression (odds ratio)). Statistically significant difference with *p* < 0.05, * with Chi squared and ^**#**^ with Fischer’s test. *p* values for each significant comparison are included in the Additional file 1: Table S1

		Professional registration			Odds ratio		
		Pod	Orth	Physio	Pod versus orth	Pod versus physio	Physio versus orth
Main working context	NHS	55.2%	45.2%	51.0%	–	–	–
	Self-employed*	28.9%	5.4%	18.4%	6.8	2.3	2.9
	PC*	2.8%	4.3%	14.3%	0.1	0.2	0.7
	PC providing NHS service^**#**^	1.1%	36.6%	2.0%	0.04	0.5	0.1
	University	2.0%	1.1%	0.0%	–	–	–
	Other^**#**^	1.1%	0.0%	6.1%	–	0.1	–
	50–50 NHS-PP	1.4%	1.1%	2.0%	–	–	–
	60–40 NHS-PP	1.1%	1.1%	0.0%	–	–	–
	70–30 NHS-PP	4.8%	5.4%	4.1%	–	–	–
	60–40 PP-NHS	0.6%	0.0%	0.0%	–	–	–
	70–30 PP-NHS	1.1%	0.0%	2.0%	–	–	–
Department	Podiatry*	82.4%	10.8%	2.0%	38.7	224	0.2
	Physiotherapy*	1.4%	9.7%	75.5%	0.1	0.005	28.8
	Orthotics*	7.6%	95.7%	6.1%	0.004	1.2	0.003
	Musculoskeletal*	32.2%	21.5%	40.8%	1.7	0.7	2.5
	Surgical appliances*	6.2%	18.3%	4.1%	0.3	1.5	0.2
	Occupational therapy	0.3%	0.0%	2.0%	–	–	–
	Rheumatology	5.6%	5.4%	2.0%	–	–	–
	CATS	4.2%	1.1%	0.0%	–	–	–
	Diabetes	2.2%	2.2%	4.1%	–	–	–
	Other	2.2%	2.2%	4.1%	–	–	–
Training undertaken since qualification	Biomechanics*	82.4%	79.6%	63.3%	1.2	2.7	0.4
	Gait analysis	57.7%	69.9%	59.2%	–	–	–
	Orthopaedics*	26.6%	38.7%	44.9%	0.6	0.4	1.3
	Footwear*	25.2%	54.8%	14.3%	0.3	2	0.1
	Podopaediatrics*	39.2%	14.0%	8.2%	3.9	7.3	0.5
	Sports*	45.7%	22.6%	55.1%	2.9	0.7	4.2
	Strength & core training*	18.5%	5.4%	38.8%	3.9	0.4	11.1
	Neurology*	16.2%	36.6%	26.5%	0.3	0.5	0.6
	Orthoses prescription*	56.0%	76.3%	32.7%	0.4	2.6	0.2
	Manipulation*	28.0%	9.7%	44.9%	3.6	0.5	7.6
	Steroid injections*	36.4%	0.0%	6.1%	–	8.8	–
	High-risk population	3.4%	2.2%	0.0%	–	–	–
	Alternative therapies	3.6%	2.2%	4.1%	–	–	–
	Diagnosis techniques	1.4%	1.1%	0.0%	–	–	–
	Surgery	3.4%	0.0%	0.0%	–	–	–
	No training	7.3%	6.5%	12.2%	–	–	–
	Other training	2.2%	2.2%	2.0%	–	–	–

*Pod* podiatrist, *physio* physiotherapist, *orth* orthotist, *NHS* National Health Service, *PC* private company, *PP* private practice, *CATS* clinical assessment and treatment service

Significant differences between the professions exist in the departments practitioners are employed in (Table 1). Each profession was mainly based in a department bearing its name (podiatry, orthotics and physiotherapy) and musculoskeletal was the second most common department for all professions (32.2%, 21.5% and 40.8%, respectively).

Biomechanics was the most common training received by all three professions (Table 1). Podiatrists were 3.9 times more likely than orthotists to have undertaken training in podopaediatrics (39.2% versus 14.0%), and 7.3 times more likely than physiotherapists (39.2% versus 8.2%). Physiotherapists were 11.0 and 2.0 times more likely than orthotists and podiatrists to have undertaken training in strength and conditioning (38.8% versus 5.4% for orthotists, and 18.5% for podiatrists). Podiatrists and physiotherapists were significantly more likely to have undertaken training related to sports (45.7% and 55.1%, respectively) and manipulation (28.0% and 44.9%, respectively) than orthotists (22.6% and 9.7% for sports and manipulation, respectively). However, orthotists were 5.0 times more likely to have received training in orthoses prescription than physiotherapists (76.3% versus 32.7%), and twice as likely as podiatrists (76.3% versus 56.0%). Podiatrists were 8.8 times more likely to undertake training in steroid injections (36.4%) than physiotherapists (6.1%) (no orthotist responders had trained in steroid injections).

### Clinical consultation

All three professions are frequently asked to decide whether a foot orthosis is appropriate (Table 2). However, physiotherapists were 4.1 times more likely than orthotists to be asked for an assessment without reference to any specific treatment (53.1% versus 21.5%, respectively) and podiatrists 2.4 times more likely than orthotists (39.5% of podiatrists). No physiotherapists received referrals that requested them to prescribe foot orthoses and orthotists were 5.0 times more likely to receive a referral requesting a foot orthosis than podiatrists (32.3% versus 10.4%).

**Table 2:** Clinical activity (percentage of responders in each profession and results of logistic regression (odds ratio)). Statistically significant difference with p < 0.05, * with Chi squared and ^**#**^ with Fischer’s test. p values for each significant comparison are included in the Additional file 1: Table S2

		Professional registration			Odds ratio		
		Pod	Orth	Physio	Pod versus orth	Pod versus physio	Physio versus orth
Referral request	Assess and decide if FO is necessary	47.1%	40.9%	42.9%	–	–	–
	Request for provision of FO*	10.4%	32.3%	0.0%	0.2	–	–
	Assessment without reference to treatment*	39.5%	21.5%	53.1%	2.4	0.1	4.1
	Self-referrals	1.4%	1.1%	0.0%	–	–	–
	Other	1.7%	4.3%	4.1%	–	–	–
% of work time providing FO/ week	< 10%*	40.6%	9.7%	87.8%	6.8	0.2	39.9
	10–50%*	39.5%	43.0%	12.2%	1.5	2.4	0.6
	51–90%*	16.8%	46.2%	0.0%	0.02	15	0.02
	91–100%	3.1%	1.1%	0.0%	–	–	–
Patients treated who have prior FO	0–25%*	57.7%	37.6%	73.5%	2.2	0.5	2.1
	26–50%*	28.3%	39.8%	18.4%	0.6	1.7	0.6
	51–75%	7.3%	11.8%	4.1%	–	–	–
	76–100%	1.7%	0.0%	4.1%	–	–	–
	Don’t know	5.3%	9.7%	0.0%	–	–	–
FO provided/ month	1–10*	38.1%	4.3%	81.6%	10.3	0.2	68.6
	11–50*	47.1%	49.5%	16.3%	1.9	4.04	0.2
	51–100*	11.8%	29.0%	2.0%	0.04	6.6	0.05
	> 100^#^	3.1%	17.2%	0.0%	0.2	–	–

*Pod* podiatrist, *physio* physiotherapist, *orth* orthotist, *FO* foot orthoses

Orthotists were far more likely to spend 51–90% of their week prescribing foot orthoses than podiatrists and physiotherapists (46.2% versus 16.8% versus 0%, respectively) (Table 2). The vast majority of physiotherapists involved in foot orthotic provision are likely to spend < 10% of their week prescribing orthoses (87.8%), compared to 49.6% for podiatrists and 9.7% for orthotists.

Orthotists were 5.0 times more likely to prescribe > 100 pairs of foot orthoses per month than podiatrists (17.2% versus 3.1%, respectively) (Table 2). Physiotherapists (81.6%) and podiatrists (38.1%) were 68.6 and 10.3 times more likely to prescribe less than 10 pairs a month than orthotists (4.3%). Only 2% of physiotherapists prescribed more than 51 pairs of foot orthoses a month.

Both podiatrists and physiotherapists had longer appointment times than orthotists, being 11.0 and 13.2 times more likely to have 45–60 min assessments (Table 3). Orthotists were 5.0 times more likely to have assessments of 15–30 min than physiotherapists and 3.0 times more likely than podiatrists. 15.1% of orthotists reported having < 15 min appointments, compared to 1.1% and 6.1% for podiatrists and physiotherapists.

**Table 3:** Details of clinical appointments/contact with patients (percentage of responders in each profession and results of logistic regression (odds ratio)). Statistically significant difference with p < 0.05, * with Chi squared and ^**#**^ with Fischer’s test. p values for each significant comparison are included in the Additional file 1: Table S3

		Professional registration			Odds ratio		
		Pod	Ortht	Physio	Pod versus orth	Pod versus physio	Physio versus orth
Time for clinical assessment	0–15 min^#^	1.1%	15.1%	6.1%	0.06	0.3	0.2
	15–30 min*	24.4%	62.4%	22.4%	0.3	1.1	0.2
	30–45 min*	42.0%	18.3%	24.5%	3.1	1.9	1.6
	45–60 min*	26.9%	3.2%	30.6%	11	0.8	13.2
	60+ min^#^	4.5%	1.1%	10.2%	4.6	0.4	10.5
	Other^#^	1.1%	0.0%	6.1%	–	0.2	–
Type of advice given on FO use	Verbal*	25.8%	21.5%	44.9%	1.3	0.4	3
	Written	5.6%	4.3%	6.1%	–	–	–
	Both*	75.9%	83.9%	34.7%	0.6	5.9	0.1
	Online	0.3%	0.0%	2.0%	–	–	–
	Video	0.3%	0.0%	2.0%	–	–	–
	Other	0.3%	0.0%	0.0%	–	–	–
% of FO supplied direct to patients	None*	60.8%	58.1%	77.6%	1	0.2	2.7
	0–30%*	28.0%	40.9%	6.1%	0.6	6.2	0.03
	31–60%	7.0%	1.1%	14.3%	–	–	–
	61–100%^#^	4.2%	0.0%	2.0%	–	0.5	–
% of patients who receive 2nd pair of FO	None*	44.8%	24.7%	75.5%	2.5	0.3	9.4
	10–30%*	43.7%	37.6%	16.3%	1.3	4	0.3
	31–70%	8.1%	15.1%	4.1%	–	–	–
	71–99%*	2.2%	16.1%	2.0%	0.2	1.7	0.2
	100%^#^	1.1%	5.4%	2.0%	0.2	0.5	0.4
Patient review	Appointment*	77.6%	57.0%	65.3%	2.6	1.8	1.4
	Telephone	15.7%	18.3%	6.1%	–	–	–
	Online	2.0%	0.0%	0.0%	–	–	–
	Patient request	0.6%	0.0%	0.0%	–	–	–
	Other	1.4%	2.2%	6.1%	–	–	–

*Pod* podiatrist, *physio* physiotherapist, *orth* orthotist, *FO* foot orthoses

In terms of dispensing orthoses, 58.1% of orthotists, 60.8% of podiatrists and 77.6% of physiotherapists never sent orthoses by post to patients (Table 3). Orthotists were 5.0 times more likely to prescribe a second pair of insoles to over 71% of their patients compared to podiatrists and physiotherapists (16.1% versus 2.2% and 2.0%, respectively). Physiotherapists (75.5%) were most likely to never offer a second pair of orthoses compared to podiatrists (44.8%) and orthotists (24.7%). Podiatrists were 2.6 times more likely than orthotists to use a clinical appointment to review the patient.

### Practitioner and patient treatment outcomes

Musculoskeletal care was the most common area of practice for all three professions (Table 4). Podiatrists and orthotists were more likely than physiotherapists to prescribe orthoses for patients with diabetes (40 and over 100 times more likely, respectively), arthritis (33.6 and almost 100 times more likely, respectively) and other high risk patients (28.0 and almost 100 times more likely, respectively). Orthotists were more likely to be involved in falls prevention (57.0% versus 30.5% for podiatrists and 8.2% for physiotherapists) and had far higher rates of involvement in paediatric (82.8%) and adult neurology practice (79.6%).

**Table 4:** Patient’s receiving foot orthoses and intended outcomes (percentage of responders in each profession and results of logistic regression (odds ratio)). Statistically significant difference with p < 0.05, * with Chi squared and ^**#**^ with Fischer’s test. p values for each significant comparison are included in the Additional file 1: Table S4

		Professional registration			Odds ratio		
		Pod	Orth	Physio	Pod versus orth	Pod versus physio	Physio versus orth
Type of patients prescribed with foot orthoses	Musculoskeletal*	92.2%	95.7%	67.3%	0.5	5.7	0.1
	Diabetes*	63.3%	94.6%	4.1%	0.9	40.5	0.002
	Arthritis*	58.8%	89.2%	4.1%	0.2	33.6	0.01
	Osteoarthritis*	72.8%	89.2%	34.7%	0.3	5.0	0.06
	Paediatric*	46.8%	82.8%	16.3%	0.2	4.5	0.04
	Neuro adult*	39.8%	79.6%	22.4%	0.2	2.3	0.07
	Neuro paediatric*	17.1%	68.8%	16.3%	0.09	1.1	0.08
	Sports	64.7%	67.7%	49.0%	–	–	–
	Other high risk*	37.3%	68.8%	2.0%	0.27	28.5	0.01
	Fall prevention*	30.5%	57.0%	8.2%	0.3	4.94	0.07
	Post-surgery	2.5%	1.1%	2.0%	–	–	–
	Other	1.1%	1.1%	4.1%	–	–	–
Practitioner’s outcomes	Pain relief*	87.4%	91.4%	63.3%	0.6	4	0.2
	Pressure relief*	47.9%	59.1%	8.2%	0.6	10.3	0.06
	Functional control	66.7%	62.4%	77.6%	–	–	–
	Accommodate deformity*	18.2%	29.0%	28.6%	0.5	0.6	1
	Stability*	12.3%	10.8%	38.8%	1.2	0.2	5.3
	Ulcer prevention*	19.0%	29.0%	4.1%	0.6	5.5	0.1
	Short term treatment*	16.5%	3.2%	38.8%	5.9	0.3	19
	Long term treatment*	24.9%	9.7%	32.7%	3.1	0.7	4.5
	Other	0.8%	1.1%	2.0%	–	–	–
Patient’s outcome	Pain reduction*	67.2%	79.6%	55.1%	0.5	1.67	0.6
	Pain free*	52.7%	41.9%	34.7%	1.5	2.1	0.4
	Return to sport*	42.9%	15.1%	38.8%	2.3	0.6	1.6
	Return to sport level	63.0%	59.1%	71.4%	–	–	–
	Return to work	8.4%	9.7%	14.3%	–	–	–
	Prevent injury*	29.4%	49.5%	8.2%	0.3	1.6	0.03
	Return to footwear	6.2%	4.3%	12.2%	–	–	–
	Prevent falls^#^	3.1%	4.3%	18.4%	0.2	0.05	1.5
	Don’t know	16.8%	22.6%	20.4%	–	–	–
	Other	1.1%	2.2%	6.1%	–	–	–

*Pod* podiatrist, *physio* physiotherapist, *orth* orthotist

Pain relief was an important outcome for 87.4%, 91.4% and 63.3% of podiatrists, orthotists and physiotherapists, respectively (Table 4). Orthotists were 10.0 times more likely to have pressure relief as an outcome than physiotherapists (59.1% versus 8.2%), and podiatrists over 10.0 times more likely than physiotherapists. Orthotists were almost twice as likely as podiatrists to have ulcer prevention as an outcome (29.0% versus 19.0%) and podiatrists were 5.5 times more likely than physiotherapists (19.0% versus 4.1%). For physiotherapists, stability was 5.0 times more likely to be an outcome than for both orthotists and podiatrists (38.8% versus 10.8% and 12.3%, respectively).

Regarding patients’ expectations of outcome, being pain free rather than reducing pain was more common for podiatrists (52.7%) than physiotherapists (34.7%) (orthotists was 41.9%). Returning to sport was an important factor for patients of podiatrists and physiotherapists compared to orthotists (2.3 and 1.6 times more likely, respectively), although returning to prior level of sport activity was not significantly different between professions. Orthotists more commonly reported an expectation to prevent injury (49.5% versus 29.4% for podiatrists and 8.2% for physiotherapists).

### Other treatments

Orthotists (96.8%) were more than 100 times more likely to prescribe footwear as well as foot orthoses compared to both podiatrists (11.8%) and physiotherapists (6.1%) (Table 5). Physiotherapists were 9.5 times more likely to have access to footwear services than orthotists. Both orthotists and podiatrists prescribe footwear to high-risk patients, such as patients with neurological problems, diabetes and arthritis, but orthotists are more likely to do this than podiatrists (Table 5). Falls prevention was a reason for footwear prescription for 29.0% of orthotists, but just 4.2% of podiatrists and no physiotherapist.

**Table 5:** Patient’s and clinical conditions associated with providing footwear (percentage of responders in each profession and results of logistic regression (odds ratio)). Statistically significant difference with p < 0.05, * with Chi squared and ^**#**^ with Fischer’s test. p values for each significant comparison are included in the Additional file 1: Table S5

		Professional registration			Odds ratio		
		Pod	Orth	Physio	Pod versus orth	Pod versus physio	Physio versus orth
Provide footwear and foot orthoses*		11.8%	96.8%	6.1%	0.004	0.5	0.002
Patients provided with footwear	Musculoskeletal*	7.6%	48.4%	2.0%	0.1	3.9	0.02
	Diabetes*	7.0%	88.2%	0.0%	0.1	–	–
	Arthritis*	5.9%	72.0%	0.0%	0.02	–	–
	Osteoarthritis*	5.6%	63.4%	0.0%	0.03	–	–
	Paediatric*	2.2%	39.8%	0.0%	0.03	–	–
	Neuro adult*	3.4%	52.7%	2.0%	0.03	1.7	0.02
	Neuro paediatric*	1.4%	48.4%	2.0%	0.01	0.7	0.02
	Sports	3.4%	7.5%	2.0%	–	–	–
	Other high risk *	5.9%	71.0%	0.0%	0.02	–	–
	Fall prevention*	4.2%	29.0%	0.0%	0.1	–	–
	Other^#^	1.1%	6.5%	2.0%	0.2	0.5	0.3
Conditions requiring footwear	Plantar fasciitis*	38.1%	22.6%	22.4%	2.1	2.1	1
	Heel pain	16.0%	20.4%	14.3%	–	–	–
	Achilles’ tendinopathy	9.8%	11.8%	20.4%	–	–	–
	OA knee pain	8.1%	7.5%	8.2%	–	–	–
	OA foot pain	15.7%	8.6%	8.2%	–	–	–
	Morton’s neuroma	10.9%	9.7%	8.2%	–	–	–
	Over-pronation	21.8%	16.1%	30.6%	–	–	–
	Other conditions	8.4%	4.3%	10.2%	–	–	–
Access to footwear service*		57.1%	7.5%	40.8%	3.9	0.4	9.5

*Pod* podiatrist, *physio* physiotherapist, *orth* orthotist, *OA* osteoarthritis

Other than for footwear, podiatrists were more likely to use other treatment approaches than orthotists and physiotherapists (Table 6). Podiatrists were 5.9 times more likely to use steroid injections than physiotherapists (34.5% versus 8.2%) and 48.3 times more likely than orthotists (1.1%). Physiotherapists were 60.8 times more likely to use mobilisation than orthotists (77.6% versus 5.4%), and 8.4 times more likely than podiatrists (32.2%). Physiotherapists and podiatrists had far greater likelihood of using taping than orthotists (75.5% and 65.3%, respectively, versus 11.8%).

**Table 6:** Non orthoses treatments provided (percentage of responders in each profession and results of logistic regression (odds ratio)). Statistically significant difference with p < 0.05, * with Chi squared and ^**#**^ with Fischer’s test. p values for each significant comparison are included in the Additional file 1: Table S6

		Professional registration			Odds ratio		
		Pod	Orth	Physio	Pod versus orth	Pod versus physio	Physio versus orth
Provides non orthoses treatments ^#^		98.0%	91.4%	98.0%	0.2	0.3	0.9
Non orthoses treatments provided	Exercise*	93.6%	61.3%	93.9%	9.2	0.9	9.7
	Footwear advice*	93.3%	82.8%	77.6%	2.9	4.02	0.7
	Footwear*	24.1%	81.7%	6.1%	0.7	4.9	0.02
	Acupuncture*	15.4%	0.0%	38.8%	–	0.3	–
	Taping*	65.3%	11.8%	75.5%	14	0.6	9.3
	Steroid injections*	34.5%	1.1%	8.2%	48.3	5.9	8.2
	Manipulation*	27.7%	3.2%	49.0%	11.5	0.4	28.8
	Mobilisation*	32.2%	5.4%	77.6%	8.4	0.1	60.8
	Trigger point therapy*	11.2%	0.0%	44.9%	–	0.2	–
	Therapeutic ultrasound*	10.9%	0.0%	44.9%	–	0.2	–
	Orthoses^#^	0.8%	6.5%	4.1%	0.1	0.2	0.6
	Surgery	3.1%	0.0%	0.0%	–	–	–
	Referral	1.1%	0.0%	0.0%	–	–	–
	Laser	2.0%	0.0%	0.0%	–	–	–
	Other^#^	2.8%	0.0%	10.2%	–	0.3	–

*Pod* podiatrist, *physio* physiotherapist, *orth* orthotist

## Discussion

The aim of this survey was to profile and compare the foot orthotic practice of podiatrists, orthotists and physiotherapists in the United Kingdom. The purpose was to provide data useful to various stakeholders. Scoping of the orthotist workforce [7] suggests ~ 350 full-time equivalents in practice and, since not all will be involved in provision of foot orthoses, our 93 responders represents a good sample of the profession. However, perhaps reflecting the sensitivities of supplying data when employed by private companies, only 36.6% of orthotists stated they were in a private company providing services to the National Health Service. This is much lower than other estimates that suggest as many as two-thirds are employed by private companies [8]. There are far more physiotherapists and podiatrists (52,500 and 13,000, respectively [9]), but only some physiotherapists will work on feet and not all of these use orthoses. Whilst the foot is the focus of podiatry practice, orthoses may not be a strategy used by all. However, data from 357 podiatrists and, although to a lesser extent, 49 physiotherapists, are reasonable samples in their own right.

Outside of those spending most of their working time in the National Health Service, podiatrists and physiotherapists had a bias towards self-employment and orthotists a bias towards private companies that provide clinical services to the National Health Service. Less than half of orthotists work directly in the National Health Service. The long standing arrangement of private companies supplying orthotists and orthoses products under contract is an acknowledged point of difference between professions [10, 11]. This may reflect the rather historical biomedical model of care, whereby orthotics (and thereby the orthotist who supplies them) were seen as “commodities” to be delivered under contract [12]. This contrasts with contemporary models of care whereby health professionals are expected to have more varied and flexible roles [13], and care is patient rather than “device” centred. Indeed, significant variations in employment context may lead to differences in autonomy, access to other services (e.g. referrals), and motivations (e.g. different service targets), although more detailed mapping of services would be required to reveal this. Several reports have pointed to the problem that seeing orthotists as commodities [11] creates, almost ‘designing-in’, variations in practice between the three professions surveyed.

There are differences between professions in the profile of time spent using foot orthoses, volume of orthoses, and patient groups treated. Orthotists spend more time providing foot orthoses and individually provide higher volumes of foot orthoses (Table 2). Nearly 50% of orthotists spend more than 50% of their week providing foot orthoses, compared to 19.9% and 0.0% for podiatrists and physiotherapists, respectively. Furthermore, far more podiatrists and physiotherapists prescribe less than 10 pairs of orthoses a month (38.1% and 81.6%, respectively) compared to orthotists (4.3%). This likely reflects greater use of other treatments by podiatrists and physiotherapists (Table 6). For example, exercise was more often advised by podiatrists and physiotherapists, and they were 3.9 and 11.1 times more likely, respectively, to have training in strength and conditioning compared to orthotists (Table 1).

Orthotists reported that orthoses, footwear and advice were commonly used, providing the focus for their treatments, while the next most common treatment was taping (11.8% of orthotists compared to 65.3% for podiatrists and 75.5% for physiotherapists). It may be that the contractual nature of orthotist work within services constrains the scope of practice. Equally, others have noted that companies supplying orthotists probably have little incentive to train staff for non-product related treatments if supply of products is key to commercially viable contracts [11].

Whilst orthotists might be more focussed on foot orthoses in terms of their time, the patients they treat span a wider range of health needs, with a higher percentage of orthotists working in all patient categories compared to podiatrists and physiotherapists (Table 4). A more focussed practice profile (foot orthoses and footwear) seems to concur with more orthotists receiving referrals that specifically request a foot orthosis. Only 21.5% receive referrals that makes no reference to treatments and thus allow them to develop a treatment strategy, compared to 53.1% for physiotherapists and 39.5% for podiatrists (Table 2). This may reflect the fact that referrers may have made a diagnosis and see the orthotist only as a specialist in the prescription of an orthotic device. It may also reflect contractual arrangements since orthotic devices, and their supply, are seen as a ‘commodity’ delivered into existing services by the orthotist [12], rather than the orthotist being an integrated part of the clinical service.

There are also differences in some of the practical aspects of practice. Orthotists, for example, typically had less time for each consultation, 62.4% had less than 30 min and 15.1% less than 15 min (compared to 1.1% for podiatrists, 6.1% for physiotherapists, Table 3). The national recommendations for orthotists is 20–40 min depending upon complexity [10]. This may be important, as orthotists see complex cases (e.g. more feet requiring pressure relief and ulcer prevention, Table 4) and prescribe footwear more often (i.e. two medical devices at the same time, Table 5) and rely on face-to-face review appointments less often (57.0% of orthotists have review appointments versus 65.3% for physiotherapy and 77.6% for podiatry, Table 3). However, given more orthotists receive referrals where diagnosis has already been determined, less time may be required. Reference has also been made to the fact that companies supplying orthotists to the National Health Service may provide clinical time at a loss and profit only from product sales [11], incentivising a higher volume of shorter appointments.

In terms of review appointments, nearly all podiatrists reviewed patients by some method (96.7%, excluding when patients request it), compared to 77.5% of both orthotists and physiotherapists (Table 3). This may speak to the difference in patient cohorts (less focus on pain in physiotherapy) and the limited time available for orthotists. It might also relate to the greater number of second pairs of orthoses provided by orthotists compared to the other professions (Table 3). Some 36.6% of orthotists provide more than 30% of their patients with a second pair of orthoses, compared to 11.4% for podiatrists and 8.1% for physiotherapists. A second pair of orthoses might negate the need to return for further pairs as it may not require a further appointment, but does not allow for adjustment of orthoses, which is common [1]. It might also reflect the fact that a company supplying an orthotist may do so at cost or a loss thus creating an incentive to supply as many orthotic products as possible [11]. Lack of review appointments is concerning and potential consequences are a lack of data on the effectiveness of orthotic intervention, especially given the complex needs of some patients (e.g. foot deformity and ulcer prevention, Table 4). Contract arrangements might not allow for review appointments since more orthotists are contracted into the National Health Service from a private company [8]. Perhaps in these cases the referrer provides the review of orthotic provision (since the referrer has decided an orthosis is needed in more cases versus podiatrists and physiotherapists). A profile of orthotist practice in the National Health Service versus that within companies contracted to the National Health Service would reveal some of these details.

Twice as many podiatrists and orthotists than physiotherapists allow orthoses to be sent directly to the patient and used without a fitting appointment (~ 40% for both versus 22.4% for physiotherapy, Table 3). It might be that podiatry and physiotherapy patients are more frequently of lower risk or orthoses fitting issues less important, or that this data relates to second pairs of orthoses being posted out. However, this would seem to be at odds with the data showing podiatrists are more likely to offer a review appointment (Table 3), and data that suggests that orthotists focus on higher risk patients (e.g. ulcer prevention, Table 4). For orthotists, it may reflect further pressure on time, since they also have the shortest appointment times and perhaps less scope for fitting appointments.

In terms of intended outcomes, pain relief was an important focus for all three professions and their patients. However, for physiotherapists, functional control was a more important outcome than pain. This is perhaps reflected in their greater focus on stability as an outcome (38.0% versus 12.3% for podiatrists and 10.8% for orthotists, Table 4), a greater desire for patients to return to sport (Table 4), and their greater training in sports, manipulation, and strength and conditioning (Table 1). This is in line with physiotherapists offering more mobilisation and manipulation than podiatrists and orthotists (Table 6). Interestingly, trigger point therapy (44.9% of physiotherapists and 11.2% of podiatrists) and acupuncture (38.8% of physiotherapists and 15.4% of podiatrists) are provided, which are in fact both pain interventions (Table 6). No orthotists offered acupuncture or trigger point therapy. Such a mixed picture points to the need for further and more nuanced data that could associate particular practices with specific patient groups and our current data does not allow for this.

Physiotherapists focus on functional control, stability and pain, and the wide range of other manual therapies they offer (Table 6), perhaps reflects the fact that they treat the whole body. Also, that they have adopted practice paradigms that consider integration of multiple body systems (a ‘system of systems’ [14]) to underpin practice. For example, they consider central pain pathways, peripheral and central roles in motor control, and upper and lower body biomechanics when treating foot problems [15]. This perhaps contrasts with podiatrists and orthotists who, in the first instance, have a greater focus on joint alignment and tissue forces local to the clinical problem. It might also explain use of foot orthoses in a different capacity, such as a short term measure in patellofemoral pain whilst using quadriceps exercises to address underlying issues with muscle function [16].

That podiatrists and orthotists had far greater focus on pressure relief than physiotherapists (47.9% and 59.1%, respectively, compared to only 8.2% for physiotherapists) perhaps speaks to their practice in high risk feet (e.g. ulcer prevention) and more complex foot conditions (e.g. for diabetes, 63.3% and 94.6%, respectively versus 4.1% for physiotherapists, Table 4). Prevention of further pathology was more important for orthotists and podiatrists (prevention of injury was 49.5% and 29.4%, respectively, versus 8.2% for physiotherapists, Table 4). Managing more complex foot conditions with orthoses would lead to a greater need for changes in footwear. For example, recommendations for offloading in diabetes advise an additional 5 mm of shoe depth to accommodate thicker insoles, which would only be achieved within non-retail footwear [17]. Managing deformity was more common for orthotists and podiatrists, and this too can necessitate non-retail footwear (e.g. in rheumatoid arthritis [18]). This perhaps explains the far greater provision of footwear by orthotists, but not the lower provision by podiatrists (e.g. 81.7% versus 24.1% for podiatrists and 6.1% for physiotherapists, Table 6).

The three professions contribute to a prevention as well as treatment agenda. Almost half of orthotists reported that prevention of injury was an expected outcome for patients (versus 29.4% for podiatrists and 8.2% for orthotists, Table 4) and 29.0% stated ulcer prevention as one of their objectives (versus 19.0% and 4.1% for podiatrists and physiotherapists, respectively, Table 4). There is some evidence that foot orthoses prevent selected injuries [19] and some plantar foot ulcers, as long as specific criteria are met (e.g. thresholds for pressure relief [20]). Some 18.4% of physiotherapists reported prevention of falls as an intended outcome for patients, and recent trials have indicated some value of orthoses as one component of a multi-faceted intervention [21, 22].

Podiatrists, orthotists and physiotherapists provide foot orthoses for a wide range of important and growing clinical groups. Meeting the national foot orthotic need requires an appropriately skilled and distributed work force. The orthotist profession is relatively small, with 350 estimated to be in practice nationally [7], compared to 13,000 podiatrists and 52,500 physiotherapists. Whilst this points to value in training more orthotists, some advising a 30–50% increase in numbers [11], the national need will be met faster and more economically by concurrently upskilling other health professionals. Upskilling should be achievable since in many cases it will build on existing knowledge related to the musculoskeletal system, pathology and biomechanics. For neuro-paediatrics, for example, 39.2% of podiatrists reported training related to children and physiotherapists are often the primary physical therapist in this area of practice [23]. Training could also consider expansion of skills in footwear prescription since it is central to an orthosis achieving the intended clinical effect [24]. Likewise, orthotists could be trained to provide a wider range of treatments, although enabling them to do so would only reduce the pool of resource for orthotic services. It might require a different supply model than the current contract structures facilitate and enable orthotist time as well as products to be valued ‘commodities’.

The rehabilitation that podiatrists, orthotists and physiotherapists offer aims to improve, maintain or restore physical strength and mobility. National Health Service England promotes the ethos that “rehabilitation is everyone’s responsibility” [25, 26], hence it does not sit with one profession. Effective rehabilitation blends the skills of many healthcare professionals to improve outcomes for individual patients. In the context of foot orthoses, our survey provides evidence that multi-profession provision of foot orthoses is already in place. Furthermore, the data indicate that foot orthoses are used as part of a programme of rehabilitation interventions. However, given differences in practice between podiatrists, physiotherapists and orthotists, there is a need to understand the wider treatment context within which foot orthoses are used. This could include how the different professions blend use of foot orthoses with the other interventions they offer, and factors that affect this (e.g. practice paradigms, and contracting of services). This would be a first step towards reducing some of the variations in practice.

There are several limitations to consider in the interpretation of this data. We were pragmatic in our sampling and this may have led to selection bias in terms of areas of practice within each profession. We are unable to determine how generalisable our sample of podiatrists, orthotists and physiotherapists is in terms of how well they relate to their wider professional groups. There were several places in the survey where we had relatively high numbers of nil responses and since the sample sizes of each profession differ the impact of this could differ between the three sets of data. This could be due to responders becoming fatigued as the questionnaire was long (60 questions).

It would have been interesting to profile the data by individual patient groups. However, we asked responders to identify any three groups they treat and thus cannot relate other data in the survey to any one specific patient group. This prevents profiling of practice by individual patient groups and thus comparison of professions at that level. This could be addressed in future work. Finally, we used regression analysis to make predictions for each aspect of practice covered by the survey. This analysis predicts the likelihood of a specific response occurring compared to another profession. However, the sample sizes for the professions are unequal and may represent their wider professional communities to different degrees. Therefore, how variation in the external validity of the three individual samples affects the comparisons of professions is not known.

## Conclusion

Podiatrists, orthotists and physiotherapists provide foot orthoses to important clinical populations in both a prevention and treatment capacity. Their working context, scope of practice and mix of clinical caseload differs significantly and is likely influenced by a range of factors. This survey described these practices and further work is needed to understand the complex factors affecting each profession. There are areas of overlap in professional practice and these could be further developed to grow a ‘national foot orthoses workforce’ that meets the growing demand on foot health services and helps meet the strategic vision for the allied health professions in the United Kingdom.

## Additional file

Additional file 1: *P* values for all statistical tests. (DOCX 34 kb)
