# Supplementary material for: National profile of foot orthotic provision in the United Kingdom, part 2: podiatrist, orthotist and physiotherapy practices
Source: J Foot Ankle Res. 2018 Mar 20;11:10. doi: 10.1186/s13047-018-0250-9 (PMC5861649; doi:10.1186/s13047-018-0250-9)
Supplement: Supplementary file 1 — P values for all statistical tests. (DOCX 34 kb) [file 13047_2018_250_MOESM1_ESM.docx]

**Table S1** p values for comparisons across the three professions and between individual professions where significant. Statistically significant difference = p<0.05, * with Chi squared and **^#^** with Fischer’s test.

|  | | **Professional registration p values** | **Logistic regression p values<** | | |
| --- | --- | --- | --- | --- | --- |
|  |  |  | **Pod versus orth** | **Pod versus physio** | **Physio versus orth** |
| **Main working context** | NHS | 0.078 | - | - | - |
|  | Self-employed* | <0.001 | <0.001 | 0.050 | 0.080 |
|  | PC* | <0.001 | <0.001 | 0.001 | 0.547 |
|  | PC providing NHS service**^#^** | <0.001 | 0.589 | 0.5 | 0.016 |
|  | University | 0.620 | - | - | - |
|  | Other**^#^** | 0.004 | - | 0.004 | - |
|  | 50-50 NHS- PP | 1 | - | - | - |
|  | 60-40 NHS- PP | 1 | - | - | - |
|  | 70-30 NHS- PP | 1 | - | - | - |
|  | 60-40 PP -NHS | 1 | - | - | - |
|  | 70-30 PP -NHS | 0.619 | - | - | - |
| **Department** | Podiatry* | <0.001 | <0.001 | <0.001 | 0.099 |
|  | Physiotherapy* | <0.001 | <0.001 | <0.001 | <0.001 |
|  | Orthotics* | 0.001 | <0.001 | 0.718 | <0.001 |
|  | Musculoskeletal* | 0.042 | 0.047 | 0.233 | 0.016 |
|  | Surgical appliances* | 0.001 | <0.001 | 0.565 | 0.031 |
|  | Occupational therapy | 0.221 | - | - | - |
|  | Rheumatology | 0.623 | - | - | - |
|  | CATS | 0.142 | - | - | - |
|  | Diabetes | 0.799 | - | - | - |
|  | Other | 0.799 | - | - | - |
| **Training undertaken since qualification** | Biomechanics* | 0.007 | 0.536 | 0.002 | 0.038 |
|  | Gait analysis | 0.1 | - | - | - |
|  | Orthopaedics* | 0.006 | 0.023 | 0.009 | 0.476 |
|  | Footwear* | <0.001 | 0.001 | 0.098 | <0.001 |
|  | Podopaediatrics* | <0.001 | <0.001 | <0.001 | 0.316 |
|  | Sports* | <0.001 | <0.001 | 0.216 | <0.001 |
|  | Strength & core training* | <0.001 | 0.004 | 0.001 | <0.001 |
|  | Neurology* | <0.001 | <0.001 | 0.079 | 0.229 |
|  | Orthoses prescription* | <0.001 | <0.001 | 0.003 | <0.001 |
|  | Manipulation* | <0.001 | <0.001 | 0.017 | <0.001 |
|  | Steroid injections* | <0.001 | - | <0.001 | - |
|  | High-risk population | 0.413 | - | - | - |
|  | Alternative therapies | 0.725 | - | - | - |
|  | Diagnosis techniques | 0.854 | - | - | - |
|  | Surgery | 0.093 | - | - | - |
|  | No training | 0.424 | - | - | - |
|  | Other training | 1 | - | - | - |

Pod = podiatrist, physio = physiotherapist, orth = orthotist. NHS = National Health Service. PC = private company, PP = private practice. CATS = clinical assessment and treatment service.

**Table S2** p values for comparisons across three professions and between individual professions where significant. Statistically significant difference = p<0.05, * with Chi squared and **^#^** with Fischer’s test.

|  | | **Professional registration p values** | **Odds ratio p values** | | |
| --- | --- | --- | --- | --- | --- |
|  |  |  | **Pod versus orth** | **Pod versus physio** | **Physio versus orth** |
| **Referral request** | **Assess and decide if FO is necessary** | 0.433 | - | - | - |
|  | **Request for provision of FO*** | <0.001 | <0.001 | - | - |
|  | **Assessment without reference to treatment*** | 0.001 | 0.002 | 0.079 | <0.001 |
|  | **Self-referrals** | 0.148 | - | - | - |
|  | **Other** | 0.620 | - | - | - |
| **% of work time providing FO/ week** | **<10%*** | <0.001 | <0.001 | <0.001 | <0.001 |
|  | **10-50%*** | 0.018 | 0.107 | 0.012 | 0.226 |
|  | **51-90%*** | <0.001 | <0.001 | 0.008 | <0.001 |
|  | **91-100%** | 0.363 | - | - | - |
| **Patients treated who have prior FO** | **0-25%*** | <0.001 | 0.001 | 0.035 | <0.001 |
|  | **26-50%*** | 0.018 | 0.029 | 0.157 | 0.011 |
|  | **51-75%** | 0.220 | - | - | - |
|  | **76-100%** | 0.127 | - | - | - |
|  | **Don’t know** | 0.051 | - | - | - |
| **FO provided/ month** | **1-10*** | <0.001 | <0.001 | <0.001 | <0.001 |
|  | **11-50*** | <0.001 | 0.759 | <0.001 | 0.001 |
|  | **51-100*** | <0.001 | <0.001 | 0.066 | 0.005 |
|  | **>100^#^** | <0.001 | <0.001 | - | - |

Pod = podiatrist, physio = physiotherapist, orth = orthotist. FO = foot orthoses.

**Table S3** p values for comparisons across three professions and between individual professions where significant. Statistically significant difference = p<0.05, * with Chi squared and **^#^** with Fischer’s test.

|  | | **Professional registration p values** | | | **Odds ratio** | | |
| --- | --- | --- | --- | --- | --- | --- | --- |
|  |  |  |  |  | **Pod versus orth** | **Pod versus physio** | **Physio versus orth** |
| **Time for clinical assessment** | **0-15 min^#^** | <0.001 | | | <0.001 | 0.133 | 0.670 |
|  | **15-30 min*** | <0.001 | | | <0.001 | 0.801 | <0.001 |
|  | **30-45 min*** | <0.001 | | | <0.001 | 0.052 | 0.255 |
|  | **45-60 min*** | <0.001 | | | <0.001 | 0.584 | <0.001 |
|  | **60+ min^#^** | 0.047 | | | 0.141 | 0.124 | 0.035 |
|  | **Other^#^** | 0.019 | | | - | 0.025 | - |
| **Type of advice given on FO use** | **Verbal*** | 0.008 | | | 0.398 | 0.006 | 0.004 |
|  | **Written** | 0.900 | | | - | - | - |
|  | **Both*** | <0.001 | | | 0.104 | <0.001 | <0.001 |
|  | **Online** | 0.221 | | | - | - | - |
|  | **Video** | 0.221 | | | - | - | - |
|  | **Other** | 1 | | | - | - | - |
| **% of FO supplied direct to patients** | **None*** | 0.027 | | | 0.849 | 0.016 | <0.001 |
|  | **1-30%*** | <0.001 | | | 0.027 | <0.001 | 0.03 |
|  | **31-60%** | 0.222 | | | - | - | - |
|  | **61-100%^#^** |  | 0.019 |  | - | 0.08  0.182 | - |
| **% of patients who receive 2^nd^ pair of FO** | **None*** | <0.001 | | | <0.001 | <0.001 | <0.001 |
|  | **10-30%*** | 0.001 | | | 0.293 | 0.001 | 0.011 |
|  | **31-70%*** | 0.048 | | | - | - | - |
|  | **71-99%*** | <0.001 | | | <0.001 | 0.626 | 0.042 |
|  | **100%^#^** | 0.033 | | | 0.366 | 0.018 | 0.589 |
| **Patient review** | **Appointment*** | <0.001 | | | <0.001 | 0.337 | 0.061 |
|  | **Telephone** | 0.146 | | | - | - | - |
|  | **Online** | 0.305 | | | - | - | - |
|  | **Patient request** | 1 | | | - | - | - |
|  | **Other** | 0.078 | | | - | - | - |

Pod = podiatrist, physio = physiotherapist, orth = orthotist. FO = foot orthoses.

**Table S4**  p values for comparisons across three professions and between individual professions where significant. Statistically significant difference = p<0.05, * with Chi squared and **^#^** with Fischer’s test.

|  | | **Professional registration p values** | **Odds ratio** | | |
| --- | --- | --- | --- | --- | --- |
|  |  |  | **Pod versus orth** | **Pod versus physio** | **Physio versus orth** |
| **Type of patients prescribed with foot orthoses** | **Musculoskeletal*** | <0.001 | 0.244 | <0.001 | <0.001 |
|  | **Diabetes*** | <0.001 | <0.001 | <0.001 | <0.001 |
|  | **Arthritis*** | <0.001 | <0.001 | <0.001 | <0.001 |
|  | **Osteoarthritis*** | <0.001 | 0.001 | <0.001 | <0.001 |
|  | **Paediatric*** | <0.001 | <0.001 | <0.001 | <0.001 |
|  | **Neuro adult*** | <0.001 | <0.001 | 0.022 | <0.001 |
|  | **Neuro paediatric*** | <0.001 | <0.001 | 0.894 | <0.001 |
|  | **Sports** | 0.068 | - | - | - |
|  | **Other high risk*** | <0.001 | <0.001 | 0.001 | <0.001 |
|  | **Fall prevention*** | <0.001 | <0.001 | 0.003 | <0.001 |
|  | **Post-surgery** | 0.800 | - | - | - |
|  | **Other** | 0.181 | - | - | - |
| **Practitioner’s outcomes** | **Pain relief*** | <0.001 | 0.289 | <0.001 | <0.001 |
|  | **Pressure relief*** | <0.001 | 0.055 | <0.001 | <0.001 |
|  | **Functional control** | 0.190 | - | - | - |
|  | **Accommodate deformity*** | 0.031 | 0.022 | 0.089 | 0.0954 |
|  | **Stability*** | <0.001 | 0.678 | <0.001 | <0.001 |
|  | **Ulcer prevention*** | 0.002 | 0.037 | 0.020 | 0.003 |
|  | **Short term treatment*** | <0.001 | 0.003 | <0.001 | <0.001 |
|  | **Long term treatment*** | 0.002 | 0.002 | 0.249 | 0.001 |
|  | **Other** | 0.755 | - | - | - |
| **Patient’s outcome** | **Pain reduction*** | 0.008 | 0.022 | 0.096 | 0.003 |
|  | **Pain free*** | 0.020 | 0.066 | 0.020 | 0.402 |
|  | **Return to sport*** | <0.001 | <0.001 | 0.588 | 0.002 |
|  | **Return to sport level** | 0.358 | - | - | - |
|  | **Return to work** | 0.441 | - | - | - |
|  | **Prevent injury*** | <0.001 | <0.001 | 0.004 | <0.001 |
|  | **Return to footwear** | 0.191 | - | - | - |
|  | **Prevent falls^#^** | <0.001 | 0.561 | <0.001 | 0.011 |
|  | **Don’t know** | 0.395 | - | - | - |
|  | **Other** | 0.51 | - | - | - |

Pod = podiatrist, physio = physiotherapist, orth = orthotist.

**Table S5**  p values for comparisons across three professions and between individual professions where significant. Statistically significant difference = p<0.05, * with Chi squared and **^#^** with Fischer’s test.

|  | | **Professional registration p values** | **Odds ratio** | | |
| --- | --- | --- | --- | --- | --- |
|  |  |  | **Pod versus orth** | **Pod versus physio** | **Physio versus orth** |
| **Provide footwear and foot orthoses*** | | <0.001 | <0.001 | 0.247 | <0.001 |
| **Patients provided with footwear** | **Musculoskeletal*** | <0.001 | <0.001 | 0.184 | <0.001 |
|  | **Diabetes*** | <0.001 | <0.001 | - | - |
|  | **Arthritis*** | <0.001 | <0.001 | - | - |
|  | **Osteoarthritis*** | <0.001 | <0.001 | - | - |
|  | **Paediatric*** | <0.001 | <0.001 | - | - |
|  | **Neuro adult*** | <0.001 | <0.001 | 0.626 | <0.001 |
|  | **Neuro paediatric*** | <0.001 | <0.001 | 0.729 | <0.001 |
|  | **Sports** | 0.117 | - | - | - |
|  | **Other high risk *** | <0.001 | <0.001 | - | - |
|  | **Fall prevention*** | <0.001 | <0.001 | - | - |
|  | **Other^#^** | 0.009 | 0.006 | 0.589 | 0.274 |
| **Conditions requiring footwear** | **Plantar fasciitis*** | 0.004 | 0.006 | 0.036 | 0.986 |
|  | **Heel pain** | 0.538 | - | - | - |
|  | **Achilles’ tendinopathy** | 0.084 | - | - | - |
|  | **OA knee pain** | 1 | - | - | - |
|  | **OA foot pain** | 0.102 | - | - | - |
|  | **Morton’s neuroma** | 0.841 | - | - | - |
|  | **Over-pronation** | 0.137 | - | - | - |
|  | **Other conditions** | 0.342 | - | - | - |
| **Access to footwear service*** | | <0.001 | 0.202 | 0.006 | 0.043 |

Pod = podiatrist, physio = physiotherapist, orth = orthotist. OA = osteoarthritis.

**Table S6** p values for comparisons across three professions and between individual professions where significant. Statistically significant difference = p<0.05, * with Chi squared and **^#^** with Fischer’s test.

|  | | **Professional registration p values** | | | **Odds ratio** | | |
| --- | --- | --- | --- | --- | --- | --- | --- |
|  |  |  |  |  | **Pod versus orth** | **Pod versus physio** | **Physio versus orth** |
| **Provides non orthoses treatments ^#^** | | 0.015 | | | 0.006 | 0.029 | 0.922 |
| **Non orthoses treatments provided** | **Exercise*** | 0.005 | | | <0.001 | 0.932 | <0.001 |
|  | **Footwear advice*** | <0.001 | | | 0.002 | 0.001 | 0.450 |
|  | **Footwear*** | <0.001 | | | <0.001 | 0.009 | <0.001 |
|  | **Acupuncture*** | <0.001 | | | - | <0.001 | - |
|  | **Taping*** | <0.001 | | | <0.001 | 0.157 | <0.001 |
|  | **Steroid injections*** | <0.001 | | | <0.001 | 0.001 | 0.064 |
|  | **Manipulation*** | <0.001 | | | <0.001 | 0.003 | <0.001 |
|  | **Mobilisation*** | <0.001 | | | <0.001 | <0.001 | <0.001 |
|  | **Trigger point therapy*** | <0.001 | | | - | <0.001 | - |
|  | **Therapeutic ultrasound*** | <0.001 | | | - | <0.001 | - |
|  | **Orthoses^#^** | 0.004 | | | 0.03 | 0.081 | 0.564 |
|  | **Surgery** | 0.101 | | | - | - | - |
|  | **Referral** | 0.613 | | | - | - | - |
|  | **Laser** | 0.305 | | | - | - | - |
|  | **Other^#^** |  | 0.007 |  | - | 0.016 | - |

Pod = podiatrist, physio = physiotherapist, orth = orthotist.
